# Structural and Spectral Investigation of a Series of Flavanone Derivatives

**DOI:** 10.3390/molecules26051298

**Published:** 2021-02-28

**Authors:** Anna Sykuła, Agnieszka Kowalska-Baron, Krystian Gałęcki, Paulina Błazińska, Elżbieta Łodyga-Chruścińska

**Affiliations:** Institute of Natural Products and Cosmetics, Faculty of Biotechnology and Food Sciences, Lodz University of Technology, Stefanowskiego Street 4/10, 90-924 Lodz, Poland; krystian.galecki@p.lodz.pl (K.G.); paulina.blazinska@dokt.p.lodz.pl (P.B.); elzbieta.lodyga-chruscinska@p.lodz.pl (E.Ł.-C.)

**Keywords:** flavanone, 2,3-dihydroflavone, Schiff base, time-dependent DFT method, binding affinity to human serum albumin, fluorescence quenching

## Abstract

Four flavanone Schiff bases (*E*)-1-(2-phenylchroman-4-ylidene)thiosemicarbazide (**FTSC**) (**1**), *N*′,2-bis((*E*)-2-phenylchroman-4-ylidene)hydrazine-1-carbothiohydrazide (**FTCH**) (**2**), (*E*)-*N’*-(2-phenylchroman-4-ylidene)benzohydrazide (**FHSB**) (**3**) and (*E*)-*N′-*(2-phenylchroman-4-ylidene)isonicotinohydrazide (**FIN**) (**4**) were synthesized and evaluated for their electronic and physicochemical properties using experimental and theoretical methods. One of them, (**2**), consists of two flavanone moieties and one substituent, the rest of the compounds (**1, 3, 4**) comprises of a flavanone-substituent system in relation to 1:1. To uncover the structural and electronic properties of flavanone Schiff bases, computational simulations and absorption spectroscopy were applied. Additionally, binding efficiencies of the studied compounds to serum albumins were evaluated using fluorescence spectroscopy. Spectral profiles of flavanone Schiff bases showed differences related to the presence of substituent groups in system B of the Schiff base molecules. Based on the theoretically predicted chemical descriptors, FTSC is the most chemically reactive among the studied compounds. Binding regions within human and bovine serum albumins of the ligands studied are in the vicinity of the Trp residue and a static mechanism dominates in fluorescence quenching.

## 1. Introduction

**Figure 1:**
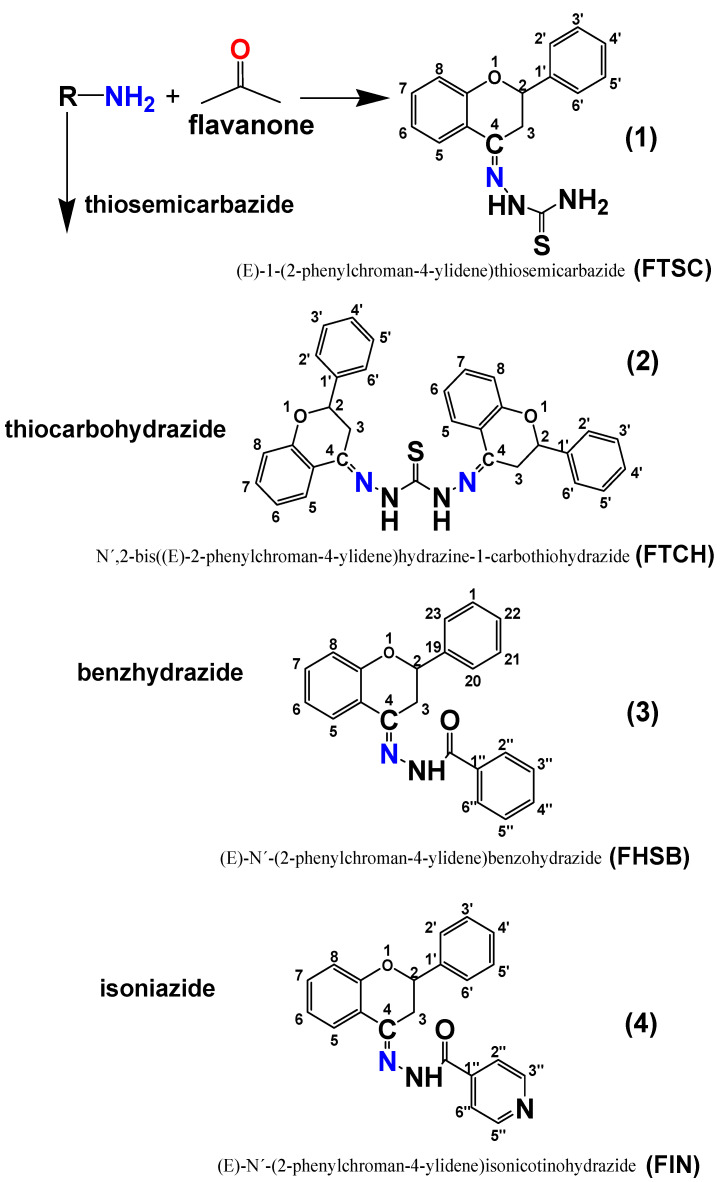
Formation of flavanone derivatives.

Schiff bases are the compounds containing azomethine group (-R^1^R^2^C=NR^3^ (R^3^ ≠ H)) [1]. Synthesis, characterization, and structure-activity relationship (SAR) of Schiff bases have been studied worldwide. Several studies showed that the presence of a lone pair of electrons in the sp^2^ hybridized orbital of the nitrogen atom of the azomethine group is of considerable chemical and biological importance [2]. They interfere in normal cell processes by the formation of a hydrogen bond between the active centers of cell constituents and the sp^2^ hybridized nitrogen atom of the azomethine group [3]. Therefore, it is interesting to study the biomolecular activity of Schiff bases. Flavanone Schiff bases of type (Figure 1) are reported in this work. They are condensation products of flavanone and thiosemicarbazide, thiocarbohydrazide, benzhydrazide or isoniazide. Flavanone Schiff base containing, in its structure, thiosemicarbazide moiety has already been described by Kállay et al. [4] and László [5]. In addition, a flavanone Schiff base containing, in its structure, the benzhydrazide group was demonstrated by Nie and Hyang [6].

Flavanone is the basic structural unit of flavanones. They are known to be precursors in the biosynthesis of all other flavanoid groups [7,8]. In various studies, flavanones show anti-proliferative, anti-inflammatory, and anti-oxidant properties therefore they have great therapeutic potential. It is suggested that natural products, including flavonoids, take part in the protective effect of fruit and vegetables against cancer. The non-substituted flavanone inhibits cell growth of cell lines A549, LLC, AGS, SK-Hepl, and HA22T [9]. Flavanone not containing hydroxyl group (OH) significantly inhibits invasion, motility, and adhesion of the cellular matrix of the cell line of the lung adenocarcinoma of A549 [10]. Non-substituted flavanones display greater antiproliferative potential in colorectal cancer cells and NH3T3 mouse fibroblasts than the flavanones with a higher number of substitutions of the OH-group [11]. Moreover, non-substituted flavanones do not exhibit antioxidant and prooxidative activities [12]. In our previous work, we showed a significant influence of thiosemicarbazide and benzohydrazide residues on the physicochemical properties and biological activity of hesperetin and naringenin.

In this paper, we used UV-Vis absorption and fluorescence spectroscopy, as well as computational simulations to characterize structural and electronic properties of the synthetized flavanone derivatives. Based on the fluorescence quenching studies, we have also assessed binding affinities of the compounds to serum albumins, since binding to albumins is one of the most important issues in the pharmacokinetics of a potential drug.

In this study, we applied both theoretical (time-dependent (TD) density functional theory (DFT) calculations) and experimental (UV-Vis and FTRIR absorption spectroscopy, fluorescence spectroscopy techniques, NMR spectroscopy) methods to determine electronic structure and properties of series of 2,3-dihydroflavone Schiff bases. One of the crucial factors which determine the bioavailability of a potential drug, and its importance in pharmacokinetics, is the estimation of the degree of binding of a drug with human serum albumin (HSA). Therefore, binding affinities of the compounds under study to HSA have been assessed based on the results of steady-state and time-resolved fluorescence measurements.

Electronic structures of the compounds under study were determined using theoretical methods, which nowadays have become increasingly applied in the first stage of studies on properties of biologically important molecules. Theoretical studies may provide very useful information based on which it is possible to assess the biological significance of newly synthetized compounds.

Density functional theory (DFT) is one of the most frequently applied theoretical approaches in the prediction of structure-related properties of flavanones and structurally similar compounds [13,14,15,16].

UV-Vis electronic spectra of medium-size molecules, such as flavanones and structurally related compounds, are usually well predicted (with relatively low computational costs) applying the time-dependent (TD) DFT formalism [17,18]. Within the framework of the TD DFT theory, the time-dependent oscillating electric field is incorporated in the ground state structure and excitation energies, oscillator strengths, and transition vectors can be determined from the linear response [19]. Since the incorporation of both solvent effects, as well as diffuse and polarization functions in the applied basis set, is mandatory for UV spectral studies for chromophores with extended π electrons [20], in this study, we have applied TD(DFT)/6-31+G(d,p)/ Polarizable Solvation Model (PCM) method to predict UV-Vis spectra of the studied Schiff bases flavanone derivatives.

The PCM model [21,22] is based on the assumption that the solute is embedded in a shape-adapted cavity within the solvent modeled as a dielectric continuum of the defined dielectric constant.

To the best of our knowledge, in the literature, there is no report on the electronic structure of flavanone derivatives. Therefore, the results presented in this study may fill this gap.

The DFT optimized structures of the new azomethine flavanone derivatives have been used to accomplish molecular docking studies with serum albumins (SAs) including human (HSA) and bovine (BSA) to establish the most preferred mode of interaction.

Additionally, in this study, fluorescence methods and docking simulations were used to provide a more detailed description of the possible mechanism of the interaction between the ligands studied (FTCH, FTSC, FIN, FHSB) and serum albumins (SAs) including human (HSA) and bovine (BSA). Serum albumins have been chosen because they are highly abundant transport proteins present in the blood plasma of mammals that play a vital role in the transportation of many substances. Binding to albumins can result in the increased solubility of hydrophobic ligands in plasma and influenced their transport to cells in vivo and in vitro. Protein—ligand binding can also modulate the ligand stability, toxicity, metabolism, free concentration, and therapeutic effect. Additionally, the ligand-albumin binding studies also could mimic those of the target cells. Hence, it is important to get information on the interaction of serum albumin and the ligands studied. In serum albumins, there are two main ligand-binding sites known as Sudlow site 1 and Sudlow site 2 [23]. Taking into account that TRP residue is located in the Sudlow site 1 and this site prefers large heterocyclic compounds, it is possible to apply fluorescence methods to study ligand binding to these proteins and to determine experimental values of the binding constants.

## 2. Results and Discussion

### 2.1. The Optimized Electronic Structures of the Studied Molecules

**Figure 2:**
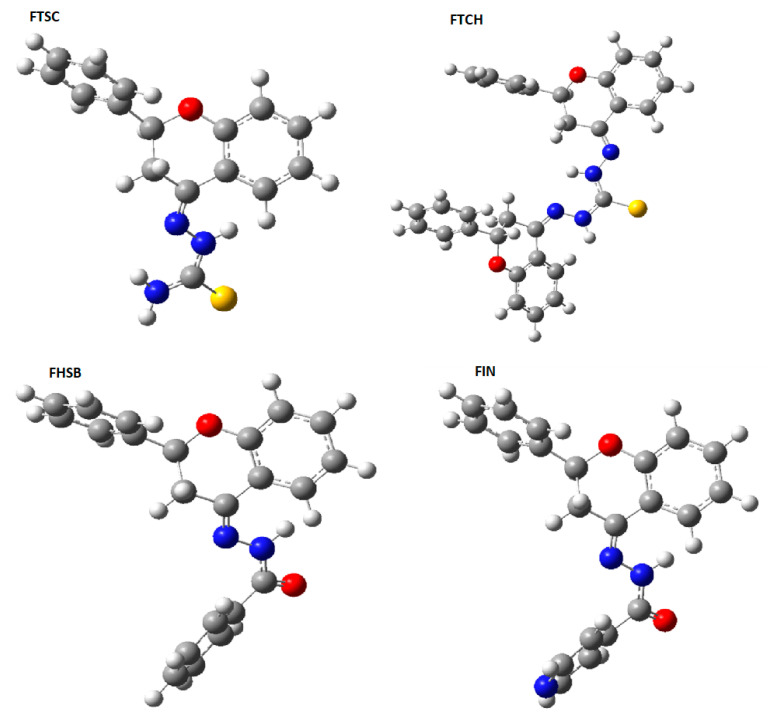
The optimized DFT (B3LYP)/6-31+G(d,p)/ Polarizable Solvation Model (PCM) (DMSO) geometries of the studied compounds.

In the molecular structures of the compounds studied, one chiral center in compounds 1–4 and two chiral centers in FTCH (C-2, according to atom numbering in Figure 1) may be noticed, therefore the studied compounds may exist in the form of enantiomers. All the possible enantiomers were optimized with the use of the PM6/DFT(B3LYP) method and the obtained results indicated that the S enantiomers are about 2–3 kcal/mol more stable than the corresponding R enantiomers (Supplementary Table S1). The DFT (B3LYP)/6-31+G(d,p)/PCM (DMSO) optimized geometries of the studied molecules are presented in Figure 2, while the optimized geometrical parameters are gathered in Supplementary Materials (Figure S1, Tables S2 and S3).

The part of the heterocyclic pyrone ring C deviates from planarity, as it can be described by the values of the C9C10C1C2 and C9C10C4C3 dihedral angles (see Tables S2 and S3 of Supplementary Materials). This deviation is caused by the discontinuity in electronic conjugation which is the result of the absence of C2-C3 double bond and the presence of substituents at C2.

Similarly, due to the electronic conjugation (delocalization) the =N-NH-C=O(S) is almost planar (see the values of C4N11N12C13, N11N12C13O14, N11N12C13S15, N11N12C13N14 in Table S2 and the values of C4N11N12N13, N11N12C13N14, N11N12C13S15 in Table S3, Supplementary Materials).

**Figure 3:**
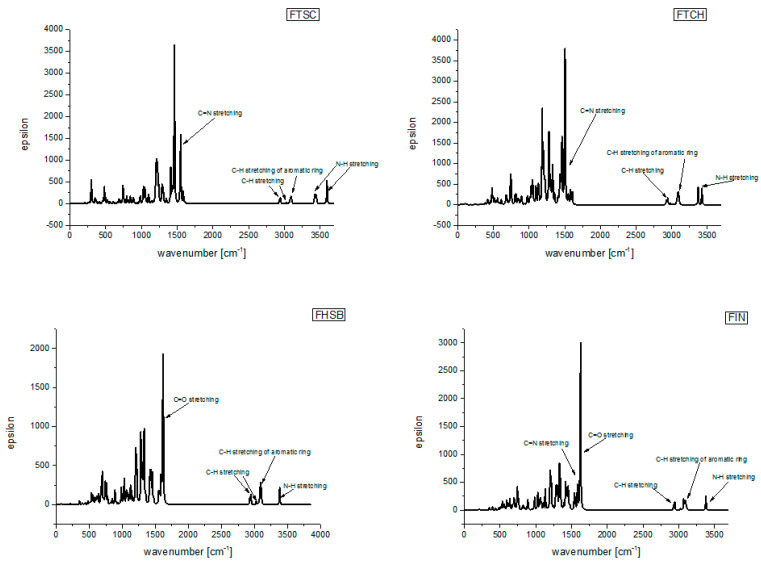
Theoretically predicted (at the density functional theory (DFT)(B3LYP)/6-31+G(d,p)/PCM(DMSO) level of theory) infra-red (IR) spectra of the studied compounds.

The theoretically predicted (at the DFT(B3LYP)/6-31+G(d,p)/PCM(DMSO) infra-red (IR) spectra are presented in Figure 3, and the calculated vibrational frequencies are gathered in Table 1, together with their experimental values.

As can be seen from Table 1, the agreement between the experimental and calculated (scaled) vibrational frequencies is quite good.

Values of quantum chemical descriptors estimated by the orbital vertical method for the DFT(B3LYP)/6-31+G(d,p) optimized compounds under study are gathered in Table 2. The calculated descriptors may provide information about the reactivity ad antioxidant properties of compounds [24]. From Table 2, it can be noticed that FTSC is characterized by the lowest value of HOMO-LUMO gap and ionization potential IP, which may indicate its higher chemical reactivity among the studied compounds.

**Figure 4:**
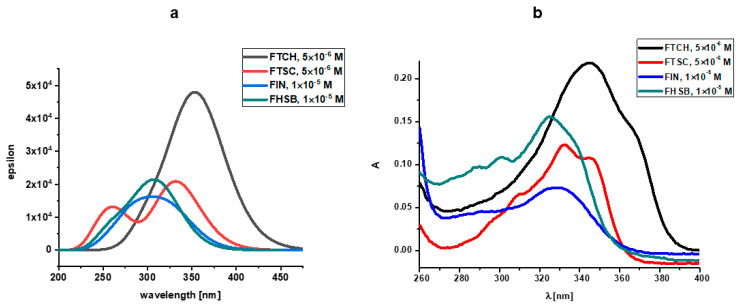
(**a**) The TD(DFT(B3LYP)/6-31+G(d,p)/PCM(DMSO) UV-Vis electronic spectra of the compounds studied (**b**) experimental UV-Vis spectra of the compounds studied in DMSO.

**Figure 5:**
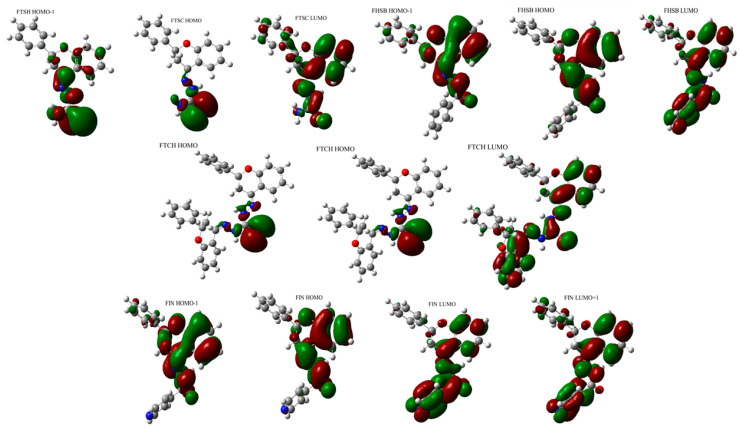
The graphical representation of orbitals participating in the lowest energy electronic transitions of FHSB, FIN, FTSC, and FTCH.

The UV-Vis electronic spectra of the compounds studied, calculated with the use of TD DFT(B3LYP)/6-31+G(d,p)/PCM(DMSO), are presented in Figure 4a together with the experimental UV-Vis spectra of the compounds studied in DMSO (Figure 4b), while the spectroscopic parameters of the electronic transitions are gathered in Table 3. The graphical representation of orbitals participating in the lowest energy electronic transitions of the compounds under study is presented in Figure 5.

The lowest energy absorption band of FHSB and FIN has a maximum located at around 320 nm. The longest wavelength absorption bands of FTSH and FTCH are red-shifted when compared to those for FHSB and FIN (Figure 3).

The results of calculations (Table 3) predicted that the lowest energy absorption band of FHSB of the energy 3.920 eV (316.29 nm) arises from a single electronic transition from HOMO to LUMO orbital and is quite intense (f = 0.3984). The S_0_→S_2_ transition of FHSB is also quite intense (f = 0.2017) and mainly involves HOMO-1→LUMO transition. The energy separation between S_0_→S_1_ and S_0_→S_2_ transition is 0.3429 eV.

The absorption maximum of FIN located around 320 nm is very broad and three transitions contribute to that band: strong (f = 0.2852) S_0_→S_1_ and S_0_→S_3_ (f = 0.1877) transitions of the energies 3.7987 eV (326.39 nm) and 4.2865 eV (289.25 nm), respectively and the weak S_0_→S_2_ transition (f = 0.0409). The latter transition is obscured by the S_0_→S_1_ and S_0_→S_2_ transitions and is very close (of 0.1572 eV) in energy to the S_0_→S_2_ transition. The S_0_→S_1_ transition of FIN is a pure HOMO-LUMO transition, while the S_0_→S_2_ and S_0_→S_3_ transitions are mainly of HOMO-1→LUMO and HOMO→LUMO+1 type, respectively.

Two bands with maxima located at about 270 nm and 330 nm may be identified in the theoretically predicted absorption spectra of FTSC. The lowest energy band arises from two close-lying S_0_→S_1_ (3.7099 eV; 334.19 nm) and S_0_→S_2_ (3.7264 eV; 332.72 nm) transitions. The S_0_→S_1_ (which is mainly of HOMO-1→LUMO type) is nearly forbidden (f = 0.0039) and obscured by the very intense (f = 0.4953) S_0_→S_2_ transition which is mainly of a HOMO-LUMO type. The two transitions: a weak (f = 0.0243) S_0_→S_3_ transition (4.0353 eV; 307.25 nm) and an intense (f = 0.1726) S_0_→S_4_ transition of the energy 4.5841 eV (270.47) contribute to the higher energy band of FTSC with the maximum around 270 nm.

The long-wavelength absorption band of FTCH with the maximum around 350 nm arises from three electronic transitions: very intense (f = 0.9372) S_0_→S_2_ transition of the energy of 3.4643 eV (357.90 nm); quite intense (f = 0.2015) S_0_→S_3_ transition (3.5803 eV; 346.30 nm); and very weak, nearly forbidden (f = 0.0033) S_0_→S_1_ located at 372.67 nm (3.3269 eV). The S_0_→S_2_ transition is mainly of the HOMO→LUMO type, whereas the S_0_→S_3_ predominantly involves a transition between and the HOMO-1 and LUMO orbitals.

Comparing the calculated UV-Vis spectrum of FHSB and FIN with the experimental ones (Figure 4), it should be noted that in the theoretical spectrum of FHSB there is a lack of vibronic structure which is seen in the experimental spectrum of that compound; and the maximum of the theoretically obtained absorption band of FIN and FHSB is about 20 nm blue-shifted as compared to the experimental spectrum.

There is a slight red shift (about 10 nm) of the maximum of the theoretical absorption band of FTCH with respect to the experimental one. Moreover, the theoretical spectrum of FTCH does not exhibit subtle vibronic structure which is seen in the experimental spectrum (see Figure 4).

The experimental spectrum of FTSC in DMSO is structured with two bands with maxima at 320 and 340 nm and a shoulder at about 310 nm; whereas in the theoretical spectrum of that compound two maxima are located at about 270 nm and 330 nm may be seen.

The discrepancies between the theoretically obtained and experimental spectra may be explained by the limitation of the applied TD DFT(B3LYP)/6-31+G(d,p)/PCM method which leads to the overestimated excitation energies [20]. Moreover, specific solute-solvent interactions, which may be responsible for the vibronic spectra seen in the experimental spectra, are not taken into account within the applied PCM solvation model.

### 2.2. Spectral Profiles of Flavanone Schiff Bases

**Figure 6:**
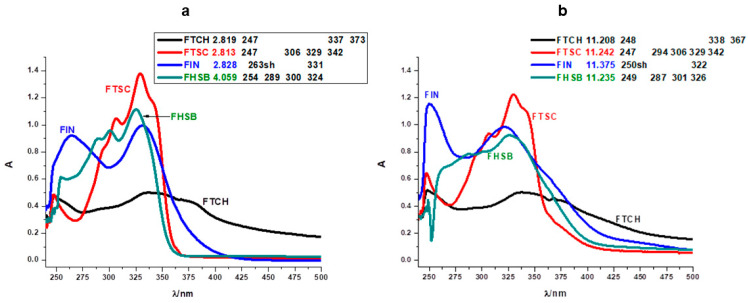
Comparison of absorption spectra of FTCH, FTSC, FIN, and FHSB in acidic (**a**) and alkaline (**b**) ranges. (Origin (Pro) software using the original files from the instrument: Perkin-Elmer Lambda 11 spectrophotometer).

Electronic spectral data are often useful in the estimation of results provided by other methods of structural examination. The UV spectra of flavanones show usually two strong absorption bands commonly referred to as band I (300–380 nm) and band II (240–280 nm) [25,26]. Band I is associated with the presence of a B-ring cinnamoyl system. Band II absorption is due to the A-ring benzoyl system. The electronic spectra of the Schiff bases (Figure 6a,b) show bands at 247–263 and 287–342 nm. The first band corresponds to the π → π* transition and the band at 287–342 nm corresponds to n → π* transition which are due to the existence of > C=N and > C=S functional groups of the Schiff bases [27]. Substitutions of the carbonyl group in the flavanone molecule with different substituents change the image of the absorption spectrum and may produce hypsochromic, bathochromic, hyperchromic, and hypochromic shifts of the absorptions (Figure 6). This phenomenon has been clearly demonstrated on hesperetin and its derivatives by Sykula and et al. [28]. The derivatives contained the same substituents such as thiosemicarbazide, isoniazid, and benzhydrazide as the compounds in this work. Hesperetin in MeOH: DMSO and DMSO solvents showed the characteristic absorption spectrum with a UV maximum at 290 nm corresponding to system A, and an inflection of low intensity at 336 nm corresponding to system B. This dependence was associated with the occurrence of π →π* transition in the aromatic ring. The spectra for the hesperetin derivatives (HHSB–with benzhydrazide, HTSC–with thiosemicarbazide, and HIN–with isoniazid) showed decreased intensity in the region connected with the A-ring benzoyl system, while the band responsible for the B-ring cinnamoyl system was more intense. In the author’s opinion, this could be caused by substituent groups in system B of the Schiff base molecules.

The assignments of the important electronic spectral bands of synthesized ligands are shown in Figure 6. Additionally, one of the tested objects was a compound (FTCH) containing two flavanone moieties and one substituent (thiocarbohydrazide), the rest of the Schiff bases—FTSC, FIN, and FHSB—consisted of one of the flavanone ring and substituent (thiosemicarbazide, isoniazid, and benzhydrazide, respectively). This difference is visible on the absorption spectrum of FTCH. The shape of band I is more evident than band II. Moreover, the spectra for all studied compounds in an acidic environment showed decreased intensity in the region of the first absorption band, while the second band was more intense (Figure 6a). This could be caused by substituent groups in system B of the Schiff base molecules, which was also reported in the work of Sykula et al. [28]. The molecular configurations indicated that the substituted groups (thiocarbohydrazide, thiosemicarbazide, isoniazide, and benzhydrazide) had an impact on π-electron transfers in the conjugated flavanone molecules. In an alkaline medium, the intensity of the bands was only changed in the case of FIN (Figure 6b). Chalcone probably was formed [29].

The spectra in Figure 6 and Figure S2 point out the different behavior of FTCH, FTSC, FIN, and FHSB. Even though they have the same basis (flavanone molecule), various substituents have an effect on the spectrum depending on the pH changes. The studied Schiff bases exhibit a band I situated at 373 nm for FTCH, 342 nm for FTSC, 331 nm for FIN, 324 for FHSB. FTCH and FTSC disclose shoulders at around 247 nm, FHSB – 254 nm, FIN – 263 nm in acidic pH while in basic region FIN and FHSB demonstrate the presence of this band located at around λ_max_ 250 nm (Figure 6a).

Due to the lack of hydroxyl groups in the flavanone ring, the basis of our modified compounds, there are no significant changes in bathochromic or hypsochromic shifts as is observed in monohydroxy flavanones [29]. In FHSB and FTSC with increasing pH, only the hypochromic effect is noticeable. This indicates that FHSB and FTSC molecules in the presence of more and more hydroxyl ions form larger, but disordered, less structured aggregates [30]. Both bands in FIN moiety influenced by pH show hypsochromic shift. The slight changes of the shift suggest interactions of FIN with hydroxyl groups. Additionally, in band I of FIN, the hyperchromic effect was observed. It could be caused by full disruption of the macromolecular aggregation [31]. In band II, hyperchromic and hypsochromic effects are present simultaneously. For FTCH in different pH, changes in the intensity and position of bands are negligible which may indicate that the structure of the molecule remains unchanged.

### 2.3. Binding Interactions with Human and Bovine Serum Albumins

**Figure 7:**
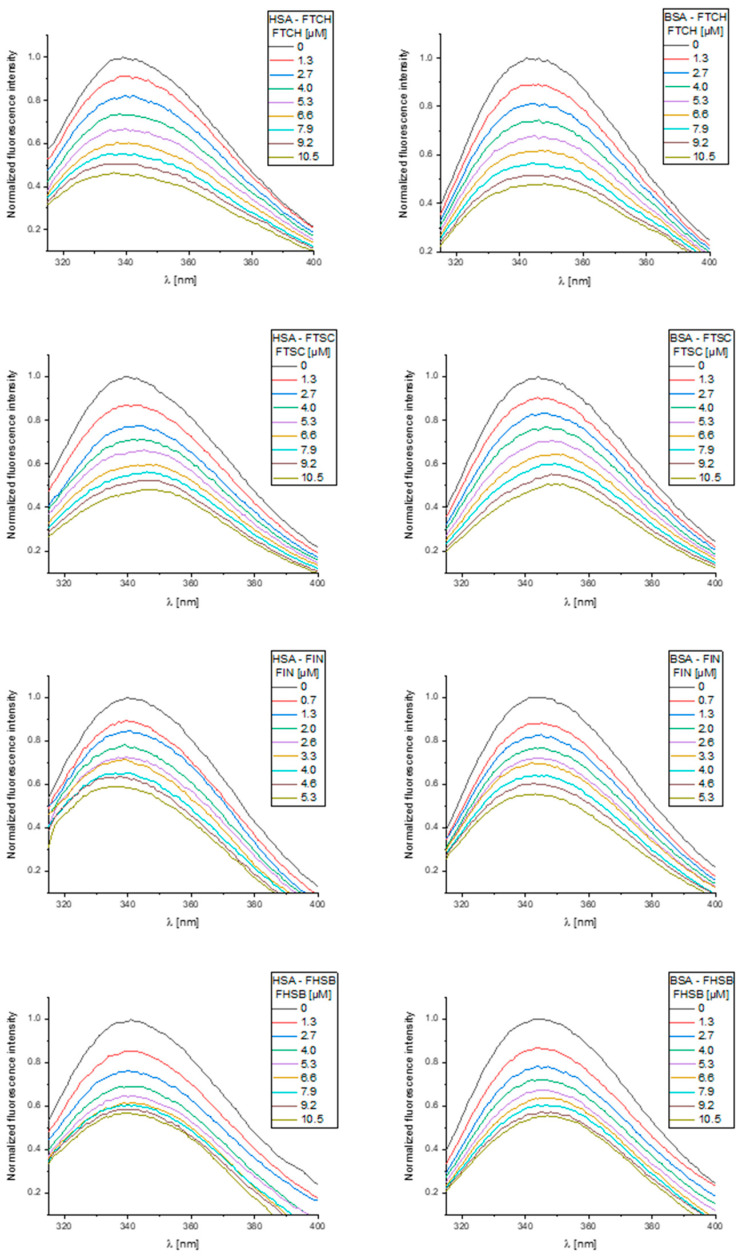
The normalized fluorescence emission spectra of human serum albumins (HSA) and bovine serum albumins (BSA) and their changes upon the increasing concentration of the ligands were studied.

The normalized steady-state fluorescence spectra of serum albumins SAs and their changes upon addition of the ligands studied are presented in Figure 7. FTCH, FTSC, FIN, and FHSB did not show intrinsic fluorescence under the experimental conditions.

From Figure 7, it may be seen that the fluorescence spectra of studied albumins showed the broad maxima located at ~340 nm (HSA) and ~345 nm (BSA), and the addition of increasing concentrations of the ligands studied caused a reduction of the fluorescence intensity (fluorescence quenching). It suggests that the binding regions of the ligands studied are in the vicinity of the Trp residue. Upon increasing concentration of FTSC, a slight red-shift (~8 nm) of the fluorescence maxima of the albumins studied may be seen and this may indicate the increase in the polarity of the nearest neighborhood of tryptophan residue. The addition of FIN to HSA and BSA and FHSB to HSA caused a slight blue shift (~5 nm) of the fluorescence maximum indicating an increased hydrophobicity of the region surrounding the tryptophan site. The observed alterations of the fluorescence spectra may also indicate that the interactions studied may involve a perturbation of the native fold [32]. FTCH did not result in any shift of the emission maxima suggesting that there was no change in the immediate environment of the tryptophan residues.

Furthermore, fluorescence quenching can occur by static and dynamic mechanisms. To distinguish between these two mechanisms, the time-resolved fluorescence measurements were conducted (supplementary data). According to Table S4 and Figure S3, upon the addition of ligands studied, a slight decrease in both the fluorescence lifetime components and in the calculated values of the average fluorescence lifetime of studied albumins was observed. Taking into account the above results, the fluorescence quenching is static and dynamic. Nevertheless, these minor changes in the fluorescence lifetime indicating that a static mechanism dominates in fluorescence quenching—that is the ground-state complex between the molecules studied—is formed.

**Figure 8:**
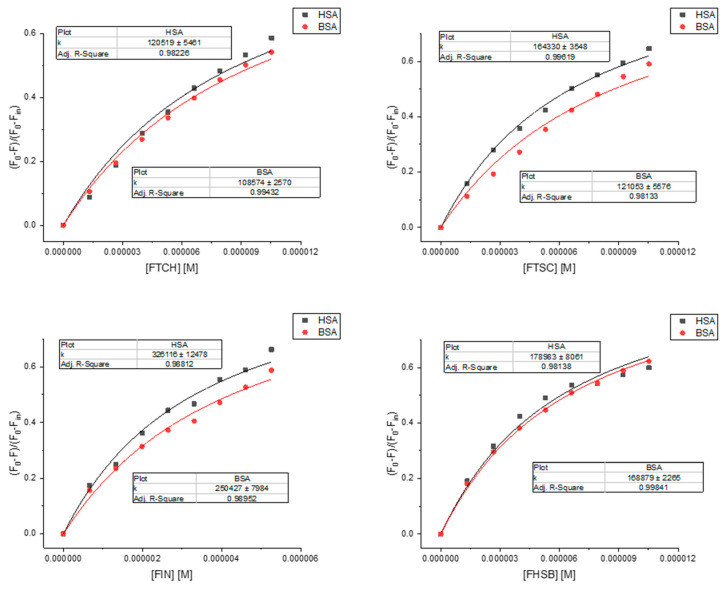
Binding curves fitted by Equation (S14).

To analyze the quenching data and determine the binding constants, the quenching model was proposed (supplementary data) and presented in Figure 8. The results of the calculations are presented in Table 4.

**Figure 9:**
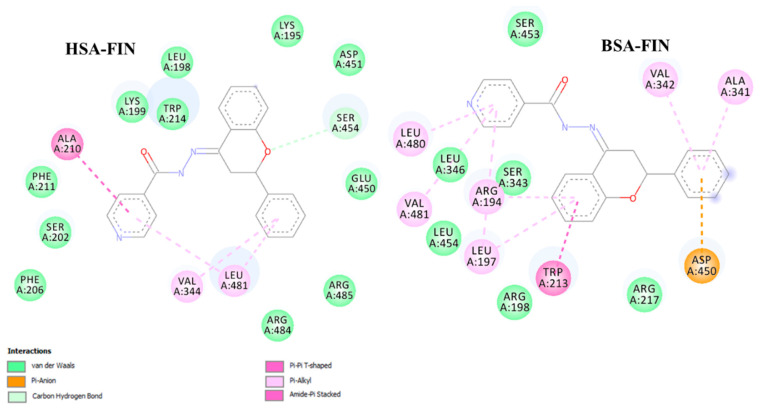
Graphical representation of the binding site for the lowest energy conformation of HSA- and BSA-FIN complex.

The binding constants that we found for the ligands studied to HSA and BSA are comparable with values for other known ligands (mostly ranged 10^4^–10^6^ M^−1^) [33]. The binding affinity to HSA and BSA is the highest for FIN. To get a deeper insight into the binding mechanism, docking simulations have been performed. We identified one high-affinity binding site, located at site 1 of subdomain IIA, close to TRP214 (HSA) and TRP213 (BSA) (Figure S5). Figure 9 illustrates the interaction of FIN with the nearby residues in the binding site of HSA and BSA (FTCH, FTSC, and FHSB are presented in Figure S6 in supplementary materials). The residues are involved in van der Waals, hydrogen bonding, and π-π interactions with the ligands studied. According to the obtained results, 4 hydrogen bonds: one in HSA-FTCH (with ASP451) and BSA-FTSC complex (with SER 343) and two hydrogen bonding in BSA-FTCH complex (with SER 343 and SER 453) may be noticed. However, the findings suggested that van der Waals interactions play a dominant role in the binding and the ligands are additionally stabilized by the π-π interactions. The results of calculations confirmed that the FIN has the highest affinity to serum albumins (Table 4) but the free energy change of binding (∆G) obtained for the lowest energy conformation from the docking simulations differ quantitatively from the experimentally obtained values. The reason for this discrepancy may be associated with the applied semiempirical energy function and/or with the neglect of solvent effects [34,35].

## 3. Materials and Methods

### 3.1. Reagents

All chemicals and solvents were purchased of analytical grade and were used without any further purification.

The racemic flavanone, thiosemicarbazide, thiocarbohydrazide, benzhydrazide, isoniazid, concentrated H_2_SO_4_, chloroform (CHCl_3_), ethanol (CH_3_CH_2_OH), and all other compounds were purchased from the Sigma-Aldrich Co. (Poznań, Poland). All reagents were of analytical quality and were used without further purification.

### 3.2. Synthesis of Ligands

Elemental analysis (C, H, N) was carried out on a EuroVector 3018 analyser (EuroVector, Milan, Italy). The melting point of the ligands was determined on an Electrothermal 9200 microscopic melting point apparatus (Cole-Parmer Ltd, Stone, Staffordshire, UK) and they were uncorrected. The IR spectra were obtained on Nicolet 6700 (Thermo-Scientific) FT-IR spectrometer (Thermo Scientific, Waltham, MA,), in the 4500–500 cm^−1^ region. Reactions were magnetically stirred and monitored by thin-layer chromatography (TLC, Merck KGaA, Darmstadt, Germany) with 0.2 mm pre-coated silica gel (8–12 μm) plates, using UV light as the visualizing agent and heating as developing agents. ^1^H NMR spectra were recorded on a Bruker AV200 (200 MHz) spectrometer (Bruker, Karlsruhe, Germany) in DMSO (dimethyl sulfoxide)-*d*_6_ with TMS (tetramethylsilane) as the internal standard. The following abbreviations were used to explain multiplicities: s = singlet, d = doublet, t = triplet, q = quartet, m = multiplet, br = broad. The ^13^C NMR spectra were obtained using a Bruker Avance III 500 spectrometer (Bruker, Karlsruhe, Germany) that operates at 125 MHz. Chemical shifts are expressed in parts per million (ppm, δ) and are referenced to DMSO-*d_6_* (δ = 39.52 ppm) as an internal standard. All spectra were taken in DMSO-*d_6_*. The formation of the new compounds was monitored by electrospray ionization mass spectrometry (ESI-MS, Bruker Daltonics GmbH, Bremen,) analysis in the negative ion mode. Before the analysis, the samples were dissolved in acetonitrile (250 µg/mL) and injected directly into a Q-Exactive Orbitrap™ tandem mass spectrometerequipped with a heated electrospray ionization (ESI) interface (HESI–II), using an injection pump and a 500 μL syringe (Thermo Scientific, Bellefonte, PA,). The injection speed was 10 μL/min. The new compounds were analyzed using full-scan MS and a subsequent parallel reaction monitoring (PRM, Thermo Scientific, Hudson, NH,) mode with a scan range from 50 to 1000 *m/z.* The capillary temperature was adjusted to 320 °C. The electrospray capillary voltage and S-lens radio frequency (RF) level were set at 4.5 kV and 50 V, respectively. Nitrogen was used as both sheath gas and auxiliary gas at a flow of 10 and 8 (arbitrary units), respectively. Ions that were selected by the quadrupole enter the higher energy collision dissociation (HCD) cell. An isolation window of 2 amu was used and precursors were fragmented by collision-induced dissociation C-trap (CID) with normalized collision energy (NCE) of 25 V. The ESI-MS/MS scan spectra (Bruker Daltonics GmbH, Bremen, Germany) were acquired with the mass resolution of 35,000 full-width at half-maximum (FWHM) at *m/z* = 100. The automatic gain control (AGC) target (the number of ions to fill C-Trap) was set at 2.0 × 10^5^ with a maximum injection time (IT) of 100 ms. Instrument control, data acquisition, and evaluation were done with the Qexactive Tune 2.1 and Thermo Xcalibur 2.2 software (Thermo Fisher Scientific, Bremen, Germany). Electronic absorption (UV–Vis) spectra were recorded with a Perkin-Elmer Lambda 11 spectrophotometer (Perkin Elmer, Ueberlingen, Germany).

(*E*)-1-(2-phenylchroman-4-ylidene)thiosemicarbazide (**FTSC 1:1**). The ligand (**1**) has been prepared according to literature procedure [28]. Yield: 0.26 g, 88%; m.p. 199–200 °C; ^1^H NMR (DMSO-*d_6_*, 200 MHz), δ= 2.82 (2H, m, 3(a), 3(e)-H); 5.23 (1H, d, J = 11 Hz, 2-H); 6.98 (2H, m, 6, 8-H); 7.36 (5H, m, 2′,3′,4′,5′,6′-H); 7.54 (1H, J = 6.6 Hz, 7-H) 8.11 (1H, s, 5-H); 8.31 (2H, d, J = 7.4 Hz, NH_2_); 10.45 (1H, bs, –NH–C=S); ^13^C NMR (DMSO-*d_6_*, 125 MHz) δ = 32.4, 76.6, 117.9, 120.6, 121.8, 125.9, 126.8, 128.6, 128.8, 131.6, 140.2, 142.4, 157.4, 179.2, ppm; IR ν_max_(cm^−1^): ν(NH_2_): 3430, ν(N-H): 3219, ν(C-H): 3137, ν(HC=N): 1598, ν(S=C-NH): 1561–1285, ν(C=S): 1217, ν(N-N):1116, UV–Vis λ_max_(nm): 332 nm; ESI-MS: *m/z* = 297.14 *m/z* [M−H]^−^; Anal. Calc. for C_16_H_15_N_3_OS: C, 64.62; H, 5.08; N, 14.13. Found: C, 64.35, 64.51; H, 5.17, 5.29; N, 14.08, 14.11.

*N*′,2-bis((*E*)-2-phenylchroman-4-ylidene)hydrazine-1-carbothiohydrazide (**FTCH 2:1**). The ligand **(2)** has been prepared according to literature procedure [28]. Yield: 0.2 g, 38%; m.p. 177–179 °C; ^1^H NMR (DMSO-*d_6_*, 200 MHz), δ= 2.96 (2H, m, 3(a), 3(e)-H); 5.29 (1H, d, J = 5.5 Hz, 2-H); 7.01 (2H, d, J = 8.4, 6, 8-H); 7.39 (5H, m, 2′, 3′,4′,5′,6′H); 7.55 (1H, s, 7-H); 8.03 (1H, d, J = 8.0, 5-H); 11.06 (1H, bs, –NH–C=S); IR ν_max_(cm^−1^): ν(N-H functions): 3283–3145, ν(HC=N): 1611, ν(S=C-NH): 1511–1221, ν(N-N):1125, ν(C=S): 748, UV–Vis λ_max_(nm): 342 nm; ESI-MS: *m/z* = 516.34 *m/z* [M−H]^−^; Anal. Calc. for C_31_H_26_N_4_O_2_S: C, 71.79; H, 5.05; N, 10.80. Found: C, 71.18; H, 5.29; N, 10.50.

(*E*)-*N′*-(2-phenylchroman-4-ylidene)benzohydrazide (**FHSB 1:1**). The ligand **(3)** has been prepared according to literature procedure [28]. Yield: 0.19 g, 55%; m.p. 203–204 °C; ^1^H NMR (DMSO-*d_6_*, 200 MHz), δ= 2.88 (2H, m, 3(a), 3(e)-H); 5.27 (1H, d, J = 9.6 Hz, 2-H); 7.04 (2H, m, 6, 8-H); 7.46 (9H, m, 7,2′,3′,4′,5′,6′,3”,4”,5”-H); 7.85 (2H, d, J = 7 Hz, 2”,6”) 8.06 (1H, d, J = 8, 5-H); 10.90 (1H, bs, –NH–C=S); ^13^C NMR (DMSO-*d_6_*, 125 MHz) δ= 33.2, 77.1, 118.2, 120.7, 121.9, 125.2, 127.0, 128.4, 128.7, 128.8, 128.9, 128.9, 132.0, 134.4, 140.3, 157.8, 180.3, ppm; IR ν_max_(cm^−1^): ν(N-H_stretch_): 3170, ν(C-H) 3066, ν(C=O): 1629, ν(C=N): 1561, ν(N-N):1116, UV–Vis λ_max_(nm): 326 nm; ESI-MS: *m/z* = 341.12 *m/z* [M−H]^−^; Anal. Calc. for C_22_H_18_N_2_O_2_: C, 77.17; H, 5.30; N, 8.18. Found: C, 76.91, 77.03; H, 5.49, 5.62; N, 7.98, 8.03.

(*E*)-*N´-*(2-phenylchroman-4-ylidene)isonicotinohydrazide (**FIN 1:1**). The ligand **(4)** has been prepared according to literature procedure [28]. Yield: 0.22 g, 65%; m.p. 201–202 °C; ^1^H NMR (DMSO-*d_6_*, 200 MHz), δ = 2.89 (2H, m, 3(a), 3(e)-H); 5.28 (1H, d, J = 12.2 Hz, 2-H); 7.05 (2H, m, 6, 8-H); 7.49 (6H, m, 7,2′,3′,4′,5′,6′-H); 7.77 (2H, d, J = 4.8 Hz, 2”,6”) 8.06 (1H, d, J = 8.2, 5-H); 8.73 (2H, d, J = 4.8 Hz, 3”,5”), 11.11 (1H, bs, –NH–C=S); ^13^C NMR (DMSO-*d_6_*, 125 MHz) δ= 33.3, 77.1, 118.2, 120.3, 122.0, 122.4, 125.3, 127.0, 128.8, 128.9; 132.4; 140.2; 150.4, 151.0; 157.7, 162.9, ppm; IR ν_max_(cm^−1^): ν(N-H_stretch_) 3217, ν(C-H): 3037, ν(C=O): 1662, ν(C=N): 1611, ν(pyridine):1533, ν(N-N):1121, UV–Vis λ_max_(nm): 328 nm; ESI-MS: *m/z* = 342.11 *m/z* [M−H]^−^; Anal. Calc. for C_21_H_17_N_3_O_2_: C, 73.45; H, 4.99; N, 12.24. Found: C, 73.02, 73.16; H, 5.09, 5.14; N, 12.21, 12.30.

### 3.3. Theoretical Methods

All calculations were performed using the Gaussian 09 [36] and GaussView 05 software package [37]. Geometry optimization of the compounds under study was performed with the use of DFT applying the hybrid B3LYP functional [38,39,40] and the 6-31+G(d,p) basis set. Our previous study has shown that this level of theory provides a quite reasonable description of structurally similar compounds [29]. Solvent effects (DMSO) were included in the calculations within the framework of the Polarizable Solvation Model (PCM). Vibrational frequency calculations at the same level of theory were performed. No imaginary vibrational frequency was found which indicated that the optimized geometries corresponded to the local minima on the potential energy hypersurface. Vibrational frequencies were scaled using the scaling factor 0.964 recommended for the applied level of theory (Computational Chemistry Comparison and Benchmark DataBase) [41].

The ionization potential (IP) and electron affinity (EA) values were calculated by orbital energy method (according to Koopmann’s theorem): IP = −E_HOMO_, EA = −E_LUMO_. Energies of the HOMO and LUMO orbitals were calculated using DFT(B3LYP)/6-31G+(d,p)/PCM (DMSO) method. Ionization potential IP is defined as the energy which is required to remove an electron, while electron affinity EA is the energy released when the electron is added. Having the IP and EA, the quantum chemical descriptors (electronegativity χ, hardness η, softness *S*, chemical potential μ, and electrophilicity index ω) were calculated according to the following Formulas (1)–(5):

(1) $$\chi =\frac{IP+EA}{2}$$

(2) $$\omega =\frac{\mu^{2}}{2\eta }$$

(3) $$S=\frac{1}{2\eta }$$

(4) $$\eta =\frac{IP-EA}{2}$$

(5) μ=−χ

Hardness η describes the resistance to charge transfer, while the inverse of hardness is given by softness *S* [42]. Electronegativity *χ* is the measure of the tendency to attract bonded electron pairs; while the electrophilicity index *ω* describes the affinity of electrons [43,44]. Chemical potential μ is the measure of escaping tendency of an electron [44].

The UV–Vis spectra and the spectroscopic parameters of the electronic transitions to the lowest excited singlet states have been obtained from the TD (nstates = 10) DFT(B3LYP)/6-31+G(d,p)/PCM(DMSO) calculations using the DFT(B3LYP)/6-31G+(d,p)/PCM(DMSO) optimized geometries.

The TD DFT (B3LYP)/6-31+G(d,p) calculations of the vertical excitations have been performed using the ground state equilibrium geometries with the linear response, non-equilibrium solvation. The reported results are for vertical transitions thus no change in geometry between the ground and excited states has been included.

### 3.4. Absorption and Fluorescence Measurements

HSA and BSA fatty acid-free were of the highest purity grade available from commercial sources (Sigma-Aldrich) and were used without further purification. The proteins (Tris buffer (5 mM, pH = 7)) were freshly prepared using high-purity water (18.2MΩ) from the Milli-Q system.

#### 3.4.1. Absorption Measurements

Absorption measurements were made using a Nicolet Evolution 300 UV-Vis spectrophotometer from Thermo Electron Corporation using the Vision Pro program. Measurements were carried out within the range of 250–600 nm, with scan speed 120 nm/min and bandwidth 1.5 nm. All measurements were performed in a standard quartz cuvette at 20 °C.

#### 3.4.2. Steady-State Fluorescence Measurements

Steady-state fluorescence measurements were performed with a FluoroMax 4 (Jobin Yvon Spex) spectrofluorimeter. All measurements were performed in a standard quartz cuvette at 20 °C. The fluorescence intensities were corrected for the absorption of the exciting light and reabsorption of the emitted light to decrease the inner filter effect using the relationship [45]:

(6) $$F_{\mathrm{cor}}=F_{\mathrm{obs}}\cdot e^{\frac{A_{\mathrm{ex}}+A_{\mathrm{em}}}{2}}$$

where F_cor_ and F_obs_ are the fluorescence intensities corrected and observed, respectively, and A_ex_ and A_em_ are the absorption of the systems at the excitation and emission wavelengths, respectively. The fluorescence intensity utilized in this study is the corrected intensity. Data analysis was performed using the software provided by Origin Pro 8.0.

#### 3.4.3. Time-Resolved Fluorescence Measurements

Fluorescence lifetime measurements were carried out at 20 °C with a FL900CDT time-correlated single-photon counting fluorimeter from Edinburgh Analytical Instruments with 295 nm excitation source (nanoLED-295) having pulse FWHM ~1.18 ns. Data acquisition and analysis were performed using the software provided by Edinburgh Analytical Instrumentation (F900). The goodness of fit was estimated by using reduced R^2^ values.

#### 3.4.4. Molecular Docking

The binding conformations of HSA and BSA and the ligands studied were predicted using the AutoDock Vina software package [46]. The crystal structures of HSA (entry codes 1H9Z) and BSA (4F5S) were retrieved from the Brookhaven Protein Data Bank [47]. The input structure of the HSA and BSA was prepared by removing co-crystallized ligands, crystal waters, and other heteroatoms and by adding Gasteiger charges and polar hydrogens using the Chimera program [48]. The input FTCH, FTSC, FIN, and FHSB structures were built and energy minimized by the Density Functional Theory (DFT) calculations with B3LYP potential and basis set 6-31G+G(d,p). First, a docking cube encompassed the whole structure of HSA and BSA, and was used throughout docking. Finally, the search spaces were restricted only in the targeted binding sites (subdomain IIa). The resulting docking solutions were subsequently clustered with a root-mean-square deviation (rmsd) tolerance of 2.0 Å and were ranked by binding energy values.

The lowest binding energy conformer was searched out of ten different conformers for each docking simulation. Discovery Studio software was also used to visualize the docking conformations [49].

## 4. Conclusions

The results of our research have shed some light on the structure and properties of the structural properties of flavanone derivatives. Flavanones, part of compounds within flavonoids, are generally known and are consumed with fruits or vegetables. However, the specific approaches to recognize the behavior of 2,3-dihydroflavone Schiff bases have rarely been studied due to their primitive structure, weak biological activity, and relatively low chemical response. Herein, we present a simple modification of 2,3-dihydroflavone structure in the form of four Schiff bases. In our work, we used flavanone as the base and four known in chemistry and medicine compounds as substituents such as thiosemicarbazide, thiocarbohydrazide, benzhydrazide, or isoniazide. First of all, the aim of the work was to demonstrate the physicochemical properties, preliminary biological behavior, and potential of 2,3-dihydroflavone Schiff bases. In this paper, we present the study in which we used UV-Vis absorption and fluorescence spectroscopies as well as computational simulations to find out differences in both the electronic structure of flavanone derivatives and in their binding efficiency to serum albumins. Such studies have not been published so far.

The applied theoretical method predicted vibrational frequencies which are in quite good agreement with experimental data. Moreover, based on the theoretical DFT(B3LYP) calculations, FTSC was characterized by the lowest value of HOMO-LUMO gap and ionization potential IP, which indicates that this compound may be the most reactive among the compounds under study. Spectral profiles of flavanone Schiff bases confirmed changes caused by substituent groups in system B of the Schiff base molecules. Research on binding interactions with human and bovine serum albumins has shown that the binding regions of the ligands studied are in the vicinity of the Trp residue. The results of time-resolved fluorescence measurements suggested that a static mechanism dominates in the tryptophan fluorescence quenching, which means that the ground-state complexes between serum albumins and the molecules studied are formed.

Summarizing, the structural changes in flavanone Schiff base rings are manifested in different spectral characteristics of the studied compounds and in their different binding affinities to serum albumins.

## Figures and Tables

**Table 1:** The DFT (B3LYP)/6-31+G(d,p)/PCM(DMSO) calculated vibrational frequencies together with their experimental values.

	ν [cm^−1^]	Scaled * ν [cm^−1]^	ν_ex_ [cm^−1^]	Description
FIN	3506	3380	3225	N-H stretching
	3236–3180	3119–3066	3035	CH stretching of aromatic ring
	3055	2945	2867	C-H stretching
	1685	1625	1650	C=O stretching
	1674	1614	1525	C=N stretching
	1650–1474	1591–1421	1450	deforming of aromatic rings
FHSB	3506	3380	very weak	N-H stretching
	3229–3180	3113–3065	3275	CH stretching of aromatic ring
	3137	3024	3165	C-H stretching
	3053	2943	3071	C-H stretching
	1675	1615		C=O stretching
	1672	1612	1621	C=N stretching
	1527–1404	1472–1353		deforming of aromatic rings
FTSC	3728	3594	3495	N-H stretching
	3572–3554	3444–3426	3433–3376	
	3222–3181	3106–3066	3215–3145	CH stretching of aromatic ring
	3138	3025	3066	C-H stretching
	3054	2944		C-H stretching
	1673	1613	2100	C=N stretching
	1649–1105	1590–1065	1500–2000	deforming of aromatic rings
FTCH	3551–3494	3424–3368	3290	N-H stretching
	3222–3180	3106–3066	3155	CH stretching of aromatic ring
	3140–3031	3027–2922	3060–2974	C-H stretching
	1667	1607	2000	C=N stretching
	1558	1502	1605	C=N stretching
	1513–1336	1459–1288	1525	deforming of aromatic rings

* Scaling factor for vibrational frequencies: 0.964 (Computational Chemistry Comparison and Benchmark DataBase).

**Table 2:** Quantum chemical descriptors (hardness (η); electronegativity (χ); chemical potential (µ); electrophilicity index (ω); softness (S)) calculated from ionization potential (IP) and electron affinity values (EA), which were estimated by orbital vertical method from the DFT(B3LYP)/6-31+G(d,p) results for the compounds under study.

Compound	HOMO [eV]	LUMO [eV]	HOMO-LUMO gap [eV]	IP [eV]	EA [eV]	η	χ	µ	ω	S
FHSB	−6.416	−2.067	4.349	6.416	2.067	2.175	4.242	−4.242	4.137	0.230
FIN	−6.359	−1.850	4.509	6.359	1.850	2.255	4.104	−4.104	3.736	0.222
FTSC	−6.173	−2.166	4.007	6.173	2.166	2.003	4.169	−4.169	4.338	0.250
FTCH	−6.197	−1.930	4.268	6.197	1.930	2.134	4.064	−4.064	3.869	0.234

**Table 3:** The TD (nstates = 10) DFT(B3LYP)/6-31+G(d,p)/PCM calculated spectroscopic parameters (transition electric dipole moment (μ); wavelength corresponding to the excitation energy (λ) and oscillator strength (f)) of the electronic transitions to the three low-lying excited singlet states of S enantiomers of FHSB, FIN, FTSC, and FTCH.

Compound	S_0_→S_1_			S_0_→S_2_			S_0_→S_3_		
	μ [D]	λ [nm]	f	μ [D]	λ [nm]	f	μ [D]	λ [nm]	f
FHSB	4.1484	316.29	0.3984	1.9310	290.84	0.2017	0.2152	275.94	0.0237
FIN	3.0648	326.39	0.2852	0.4040	300.26	0.0409	1.7873	289.25	0.1877
FTSC	0.0433	334.19	0.0039	5.4252	332.72	0.4953	0.2457 1.5365	307.25 270.47	0.0243 0.1726
FTCH	0.0405	372.67	0.0033	11.0420	357.90	0.9372	2.2967	346.30	0.2015

**Table 4:** Binding constant and Gibbs Energy for FTCH, FTSC, FIN, and FHSB to HSA and BSA.

		K_a_ [M^−1^]	∆G [kcal/mol] *	
		Experiment	Experiment	Docking
HSA	FTCH	(1.2 ± 0.1) × 10^5^	−6.8	−8.8
	FTSC	(1.6 ± 0.1) × 10^5^	−7.0	−8.9
	FIN	(3.3 ± 0.1) × 10^5^	−7.3	−9.5
	FHSB	(1.8 ± 0.1) × 10^5^	−7.0	−8.9
BSA	FTCH	(1.1 ± 0.1) × 10^5^	−6.8	−8.9
	FTSC	(1.2 ± 0.1) × 10^5^	−6.8	−8.5
	FIN	(2.5 ± 0.1) × 10^5^	−7.2	−9.4
	FHSB	(1.7 ± 0.1) × 10^5^	−7.0	−9.0

* Defined as ∆G = −RTlnK_a_ (T = 293.15 K).

## Data Availability

The data presented in this study are available on request from the corresponding author.

## Supplementary Materials

The following are available online, Table S1: The DFT(B3LYP)/6-31+G(d,p)/PCM(DMSO) calculated electronic energy values of the optimized structures. Figure S1: The DFT(B3LYP)/6-31+G(d,p)/(PCM=DMSO) optimized geometries of the studied compounds together with atom numbering., Table S2: The DFT(B3LYP)/6-31+G(d,p)/(PCM=DMSO) optimized geometrical parameters of the studied compounds: FHSB, FIN and FTSC, Table S3: The DFT(B3LYP)/6-31+G(d,p)/(PCM=DMSO) optimized geometrical parameters of FTCH, Figure S2: Absorption spectra of 2.5×10^−5^ M solution of FHSB, 5×10^−5^ M solutions of FTSC and FTCH and 1×10^−4^ M solution of FIN at different pH, Table S4: The changes in fluorescence lifetimes (τ_1_ [ns], τ_2_ [ns], τ_3_ [ns], <τ> [ns]) and their fractional fluorescence of HSA and BSA upon addition of the compounds studied, Figure S3: The kinetics of the fluorescence decay of HSA (left) and BSA (right) in the absence and presence of the ligands studied, Figure S4: Plots of integrated fluorescence intensity vs concentration of the ligands studied fitted by monoexponential function for determination of $F_{\infty }$ value (as y0), Figure S5: Graphical representation of the lowest energy conformation of HSA (left) and BSA (right) complex with FTCH (red), FTSC (yellow), FIN (cyan), and FHSB (blue). Figure S6. Graphical representation of the binding site for the lowest energy conformation of HSA–and BSA–FTCH, FTSC, FHSB complexes. Figure S7. ^1^H NMR spectrum of compound FTSC (1). Figure S8. ^13^C NMR spectrum of compound FTSC (1). Figure S9. ^1^H NMR spectrum of compound FTCH (2). Figure S10. ^1^H NMR spectrum of compound FHSB (3). Figure S11. ^13^C NMR spectrum of compound FHSB (3). Figure S12. ^1^H NMR spectrum of compound FIN (4). Figure S13. ^13^C NMR spectrum of compound FIN (4).

## Sample Availability

Samples of the compounds (FTSC, FHSB and FIN) are available from the authors.
