# Supplementary material for: Structural and Spectral Investigation of a Series of Flavanone Derivatives
Source: Molecules. 2021 Feb 28;26(5):1298. doi: 10.3390/molecules26051298 (PMC7957484; doi:10.3390/molecules26051298)
Supplement: Supplementary file 1 [file molecules-26-01298-s001.pdf]

Supplementary materials

Synthesis and characterisation of novel 2,3-dihydroflavone Schiff bases for bioapplications

Anna Sykuła*, Agnieszka Kowalska-Baron*, Krystian Gałęcki, Paulina Błazińska and Elżbieta Łodyga-Chruścińska

Institute of Natural Products and Cosmetics, Faculty of Biotechnology and Food Sciences, Lodz University of Technology, Stefanowskiego Street 4/10, 90-924 Lodz, Poland; anna.sykula@p.lodz.pl (A.S.); agnieszka.kowalska-baron@p.lodz.pl (A.K.B.); krystian.galecki@p.lodz.pl (K.G.); paulina.blazinska@dokt.p.lodz.pl (P.B.); elzbieta.lodyga-chruscinska@p.lodz.pl (E.Ł.Ch.)

* Correspondence: anna.sykula@p.lodz.pl; Tel.: +48-4263-134-17 (A.S.):, agnieszka.kowalska-baron@p.lodz.pl; Tel.: +48-4263-134-10 (A.K.B.)

Table S1 The DFT(B3LYP)/6-31+G(d,p)/PCM(DMSO) calculated electronic energy values of the optimized structures.

	S [a.u]	R [a.u]
FHSB	-1109,19807780	-1109.19538088
FIN	-1125,23352725	-1125,23083335
FTSC	-1256.46719972	-1256.46455854
FTCH	-1964,67285925	-1964,66846252

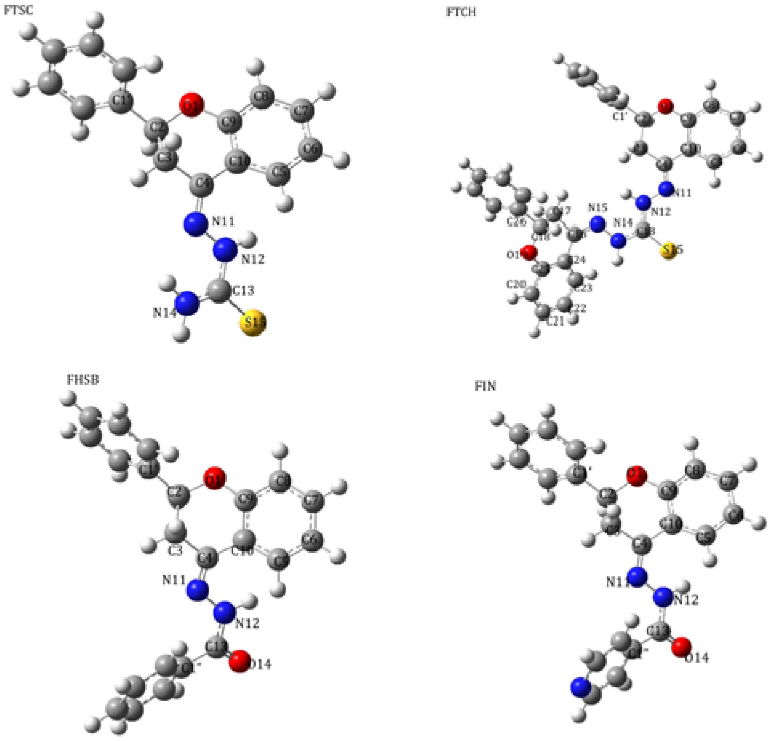

Figure S1. The DFT(B3LYP)/6-31+G(d,p)/(PCM=DMSO) optimized geometries of the studied compounds together with atom numbering.

Table S2 The DFT(B3LYP)/6-31+G(d,p)/(PCM=DMSO) optimized geometrical parameters of the studied compounds: FHSB, FIN and FTSC.

bond length [Å]	FHSB	FIN	FTSC
C2C1′	1.51192	1,51193	1,51202
O1C2	1.45176	1,45187	1,45191
C2C3	1.53590	1,53617	1,53617
C3C4	1.50832	1,50804	1,50774
C4C10	1.47699	1,47637	1,47470
O1C9	1.36446	1,36400	1,36359
C9C10	1.41684	1,41678	1,41694
C5C10	1.41266	1,41278	1,41303
C5C6	1.39131	1,39107	1,39080
C6C7	1.40159	1,40171	1,40188
C7C8	1.39142	1,39129	1,39116
C8C9	1.40141	1,40142	1,40143
C4N11	1.29279	1,29280	1,29379
N11N12	1.37314	1,37431	1,37118
N12C13	1.37477	1,36865	1,36358
C13O14	1.23879	1,23637	-
C13C1″	1.49497	1,50144	-
C13N14	-	-	1,33923
C13S15	-	-	1,70275
**torsion angle [°]**	**FHSB**	**FIN**	**FTSC**
C2O1C9C10	-6,825	-6,649	-6,541
C9C10C4C3	-23,101	-23,298	-23,122
O1C2C1′C2′	-50,383	-50,551	-50,727
O1C2C1′C6′	131,512	131,280	131,127
C3C2C1′C2′	71,877	71,738	71,526
C3C2C1′C6′	-106,227	-106,420	-106,620
C2C3C4N11	-123,953	-123,722	-124,240
C9C10C4N11	152.442	-152,161	152,646
C3C4N11N12	172.558	172,346	172,786
C10C4N11N12	-2.881	-3,009	-2,887
C4N11N12C13	-178.467	-179,864	179,168
N11N12C13O14	161,775	164,606	-
N11N12C13C1″	-20,141	-17,240	
N11N12C13N14	-	-	-8,143
N11N12C13S15	-	-	173,076

Table S3 The DFT(B3LYP)/6-31+G(d,p)/(PCM=DMSO) optimized geometrical parameters of FTCH.

FTCH	bond length [Å]	FTCH	torsion angle [°]
C2C1′	1,51150	C2C3C4N11	-156,518
O1C2	1,44708	C9C10C4N11	-175,659
C2C3	1,53371	C3C4N11N12	-0,419
C3C4	1,51433	C10C4N11N12	176,717
C4C10	1,47294	C4N11N12N13	179,294

C5C10	1,41044	N11N12C13N14	178,161
O1C9	1,37004	N11N12C13S15	-3,100
C9C10	1,41063	O1C2C1′C2′	-54,273
C5C6	1,38938	O1C2C1′C6′	126,776
C6C7	1,40445	C3C2C1′C2′	68,057
C7C8	1,39155	C3C2C1′C6′	-110,894
C8C9	1,40081	C2O1C9C10	-26,421
C4N11	1,29463	C9C10C4C3	1,733
N11N12	1,36291	N12C13N14N15	-8,671
N12C13	1,35554	C13N14N15C16	-179,373
C13N14	1,37060	N14N15C16C17	173,353
C13S15	1,69075	N15C16C17C18	-124,746
N14N15	1,36989	C16C17C18O19	-58,724
N15C16	1,29417	C16C17C18C25	-179,702
C16C17	1,50782		
C17C18	1,53546		
C18O19	1,45167		
O19C25	1,36360		
C24C25	1,41750		
C16C24	1,47373		
C20C25	1,40124		
C20C21	1,39116		
C21C22	1,40189		
C22C23	1,39064		
C23C24	1,41333		

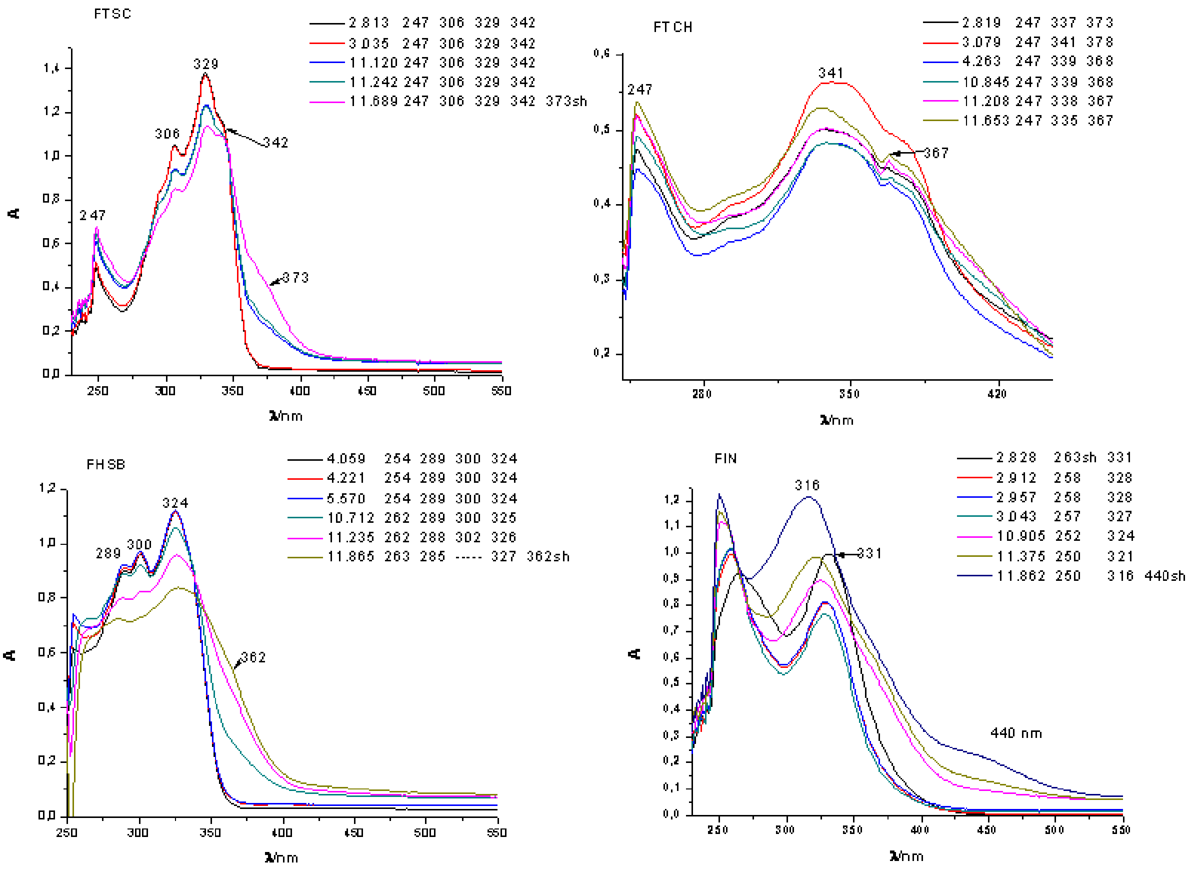

Figure S2. Absorption spectra of 2.5×10^{-5} M solution of FHSB, 5×10^{-5} M solutions of FTSC and FTCH and 1×10^{-4} M solution of FIN at different pH.

Table S4. The changes in fluorescence lifetimes (τ_1 [ns], τ_2 [ns], τ_3 [ns], $<\tau>$ [ns]) and their fractional fluorescence of HSA and BSA upon addition of the compounds studied.

		τ_1 [ns]	f_1 [%]	τ_2 [ns]	f_2 [%]	τ_3 [ns]	f_3 [%]	$<\tau>$* [ns]	$<\tau>/<\tau_0>$	R^2
	HSA	0.23±0.04	7.33	2.84±0.13	31.30	6.85±0.10	61.38	5.11±0.10	1.00	1.09
	DMSO	0.05±0.03	7.95	2.51±0.10	26.58	6.67±0.07	65.47	5.05±0.07	0.99	1.06
	5 µM FTCH	0.20±0.04	9.16	2.71±0.12	30.13	6.94±0.09	60.71	5.05±0.10	0.99	1.19
	10 µM FTCH	0.17±0.03	14.27	3.08±0.11	38.51	7.66±0.15	47.22	4.83±0.12	0.94	1.10
	5 µM FTSC	0.15±0.05	8.20	2.32±0.10	29.07	6.56±0.07	62.73	4.80±0.08	0.94	1.16
HSA	10 µM FTSC	0.12±0.04	10.13	2.27±0.08	31.38	6.70±0.08	58.49	4.64±0.08	0.91	1.09
	3 µM FIN	0.36±0.04	10.83	3.01±0.15	35.41	7.09±0.14	53.77	4.92±0.13	0.96	1.13
	5 µM FIN	0.12±0.04	12.08	2.59±0.10	32.15	6.91±0.10	55.77	4.70±0.09	0.92	1.13
	5 µM FHSB	0.24±0.03	10.21	2.61±0.10	36.58	7.13±0.10	53.22	4.77±0.09	0.93	1.14
	10 µM FHSB	0.30±0.03	13.30	2.91±0.11	40.16	7.56±0.14	46.54	4.73±0.11	0.92	1.05

	BSA	-	-	1.69±0.16	6.10	6.50±0.03	93.90	6.21±0.04	1.00	1.08
	DMSO	-	-	2.15±0.17	8.69	6.53±0.04	91.31	6.15±0.05	0.99	1.05
	5 µM FTCH	-	-	1.64±0.10	9.45	6.53±0.03	90.55	6.07±0.04	0.98	1.18
	10 µM FTCH	-	-	1.13±0.06	9.71	6.30±0.02	90.29	5.79±0.03	0.93	1.22
	5 µM FTSC	-	-	1.46±0.09	8.97	6.21±0.03	91.03	5.79±0.03	0.93	1.14
BSA	10 µM FTSC	-	-	1.53±0.08	11.85	6.18±0.03	88.15	5.63±0.04	0.91	1.14
	3 µM FIN	-	-	1.39±0.08	9.89	6.21±0.03	90.11	5.73±0.03	0.92	1.16
	5 µM FIN	-	-	1.56±0.06	15.94	6.17±0.03	84.06	5.44±0.04	0.88	1.25
	5 µM FHSB	-	-	1.25±0.06	10.83	6.13±0.03	89.17	5.60±0.03	0.90	1.19
	10 µM FHSB	-	-	1.51±0.06	14.16	6.21±0.03	85.84	5.55±0.03	0.89	1.19

*Defined as $\langle\tau\rangle=\Sigma f_i \cdot \tau_i$. Instrument parameters: Counts = 2000, Time range = 50 ns, Channels = 1024, Time per channel = 49 ps, λ_{ex}=295 nm, λ_{em}=340 nm, FWHM ~1.18ns.

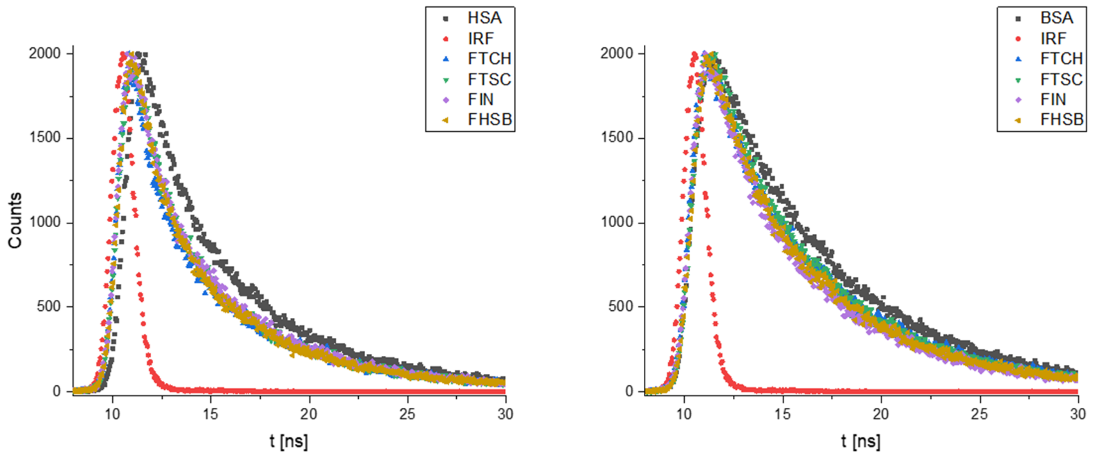

Figure S3. The kinetics of the fluorescence decay of HSA (left) and BSA (right) in the absence and presence of the ligands studied.

Quenching Model

For the one binding site:

$$[P] + [L] \overset{k}{\leftrightarrow} [PL] \tag{S1}$$

where: [P] – the concetration of free protein, [L] – the concentration of free ligand, [PL] – the concentration of complex, k – binding constant

And:

$$[P] = [P]_{an} - [PL] \tag{S2}$$

$$[L] = [L]_{an} - [PL] \tag{S3}$$

where: $[P]_{an}$ – the analitycal concentration of protein, $[L]_{an}$ – the analitycal concentration of ligand.

That, the binding constant is equal to:

$$k = \frac{[PL]}{[P]\cdot[L]} = \frac{[PL]}{([P]_{an}-[PL])\cdot([L]_{an}-[PL])} \tag{S4}$$

$$k \cdot [PL]^2 - (k \cdot [P]_{an} + k \cdot [L]_{an} + 1) \cdot [PL] + k \cdot [P]_{an} \cdot [L]_{an} = 0 \tag{S5}$$

After some rearrangements, the concentration of complex is equal to:

$$[PL] = \frac{1+k\cdot[P]_{an}+k\cdot[L]_{an}-\sqrt{-4\cdot k^2\cdot[P]_{an}\cdot[L]_{an}-(1+k\cdot[P]_{an}+k\cdot[L]_{an})^2}}{2\cdot k} \tag{S6}$$

Emission of light can be expressed by the following equation:

$$F_i = \alpha \cdot \sum_i \varepsilon_i \cdot \Phi_i \cdot c_i \tag{S7}$$

where: F_i – the fluorescence intensity of i, α – apparatus factors, ε_i – molar extinction coefficient of i, Φ_i – fluorescence quantum yield of i, c_i – concentration of i.

In our studies:

$$F_L = \alpha\{\varepsilon_{Trp} \cdot \Phi_{Trp} \cdot [P] + \varepsilon'_{Trp} \cdot \Phi'_{Trp} \cdot [PL] + \varepsilon_L \cdot \Phi_L \cdot [L]\} \tag{S8}$$

where: Φ'_{Trp} - fluorescence quantum yield of Trp changed by the interaction of the ligand

From the experimental conditions:

$$\Phi_{[L]} = 0 \tag{S9}$$

Therefore:

$$F_L = \alpha \cdot \varepsilon_{Trp} \cdot \Phi_{Trp} \cdot ([P]_{an} - [PL]) + \alpha \cdot \varepsilon'_{Trp} \cdot \Phi'_{Trp} \cdot [PL] \tag{S10}$$

In the absence of the quencher:

$$F_0 = \alpha \cdot \varepsilon_{Trp} \cdot \Phi_{Trp} \cdot [P]_{an} \tag{S11}$$

and, the infinite fluorescence intensity (Figure S4) is:

$$F_\infty = \alpha \cdot \varepsilon'_{Trp} \cdot \Phi'_{Trp} \cdot [P]_{an} \tag{S12}$$

Therefore, eq. 10 has the form:

$$F_L = F_0 + \left(\frac{F_\infty}{[P]_{an}} - \frac{F_0}{[P]_{an}}\right) \cdot [PL] \tag{S13}$$

Finally, after simple rearrangements, the data may be fitted by eq. (14) of the following form:

$$\frac{F_0 - F_L}{F_0 - F_\infty} = \frac{[PL]}{[P]_{an}} = \frac{1 + k \cdot [P]_{an} + k \cdot [L]_{an} - \sqrt{-4 \cdot k^2 \cdot [P]_{an} \cdot [L]_{an} - (1 + k \cdot [P]_{an} + k \cdot [L]_{an})^2}}{2 \cdot k \cdot [P]_{an}} \tag{S14}$$

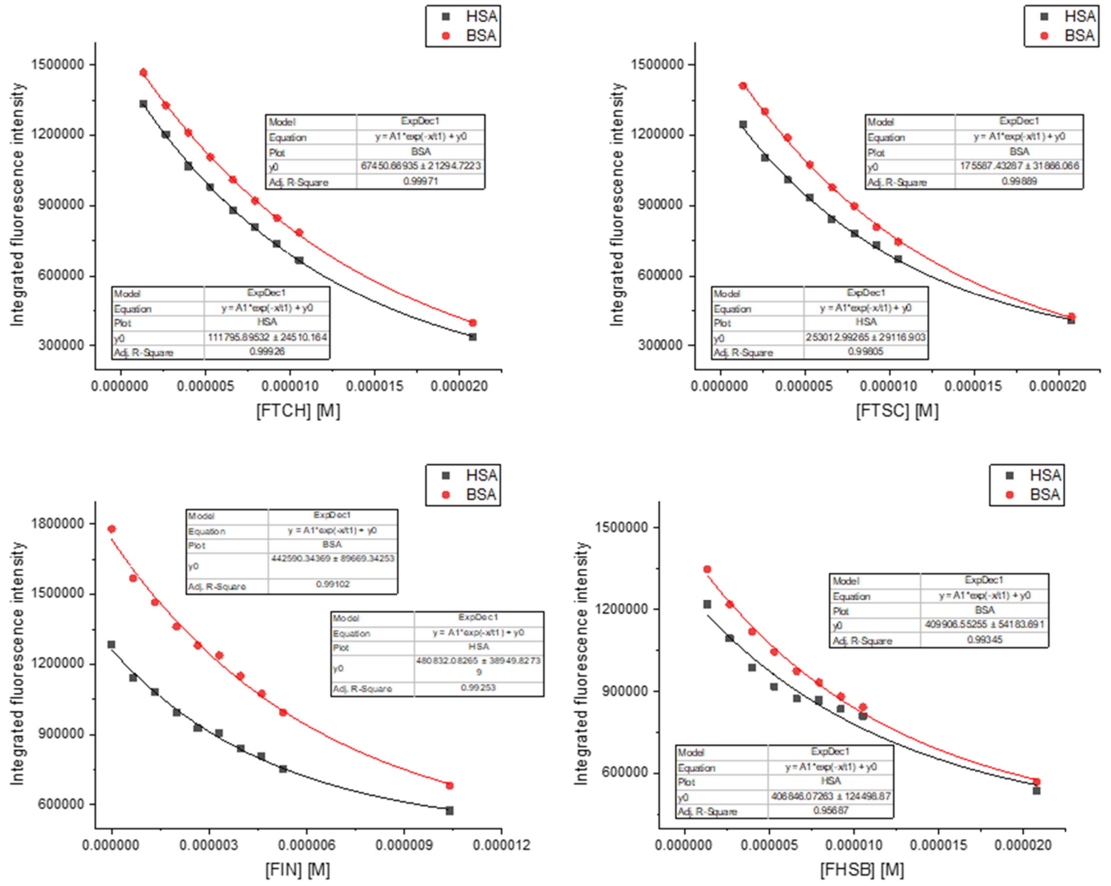

Figure S4. Plots of integrated fluorescence intensity vs concentration of the ligands studied fitted by monoexponential function for determination of F_{∞} value (as y0).

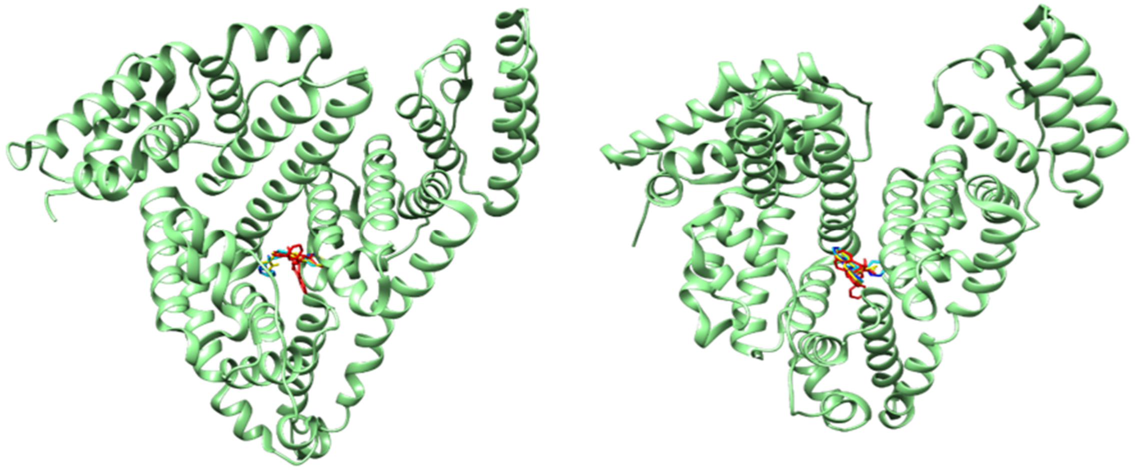

Figure S5. Graphical representation of the lowest Energy conformation of HSA (left) and BSA (right) complex with FTCH (red), FTSC (yellow), FIN (cyan) and FHSB (blue).

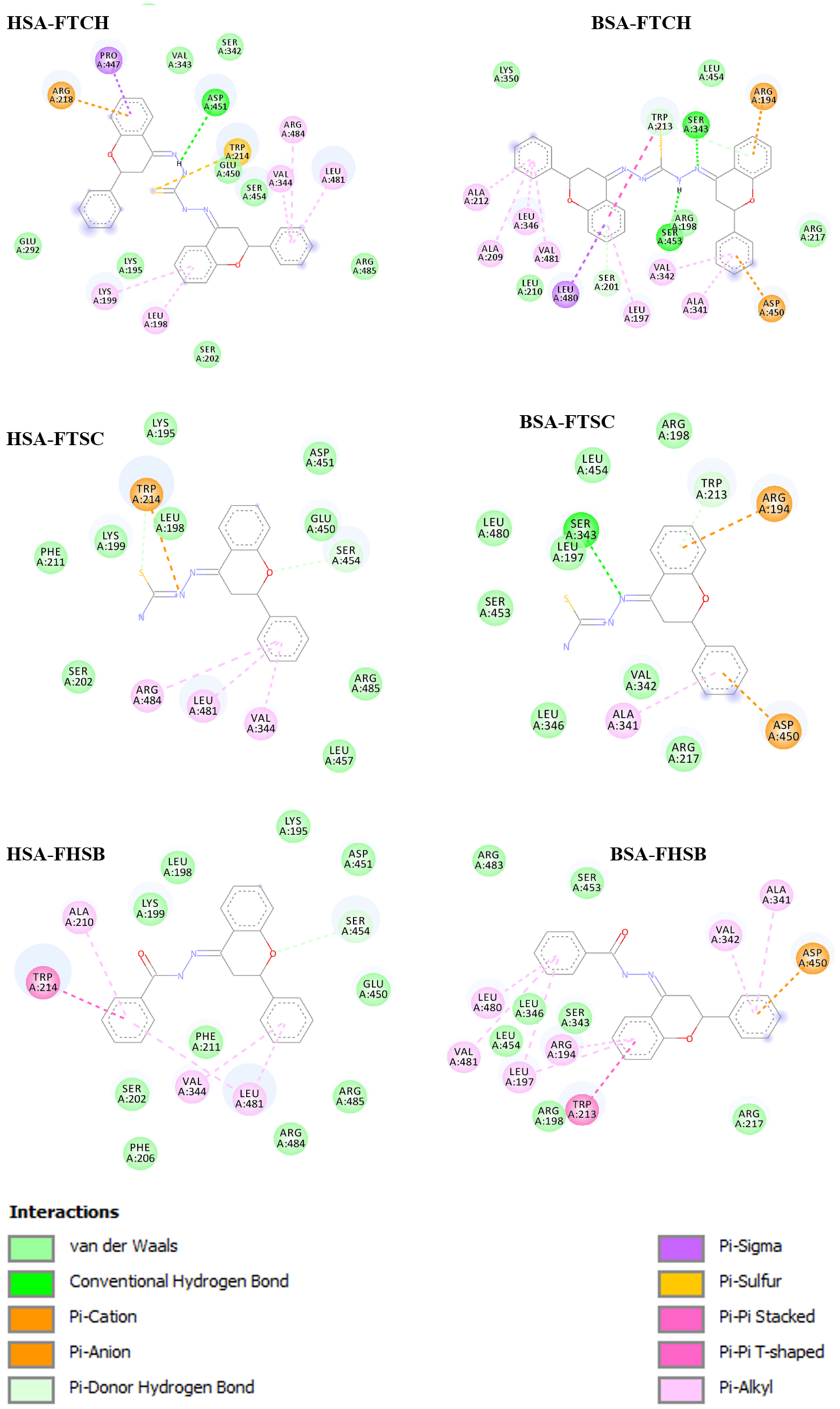

Figure S6. Graphical representation of the binding site for the lowest energy conformation of HSA – and BSA – FTCH, FTSC, FHSB complexes.

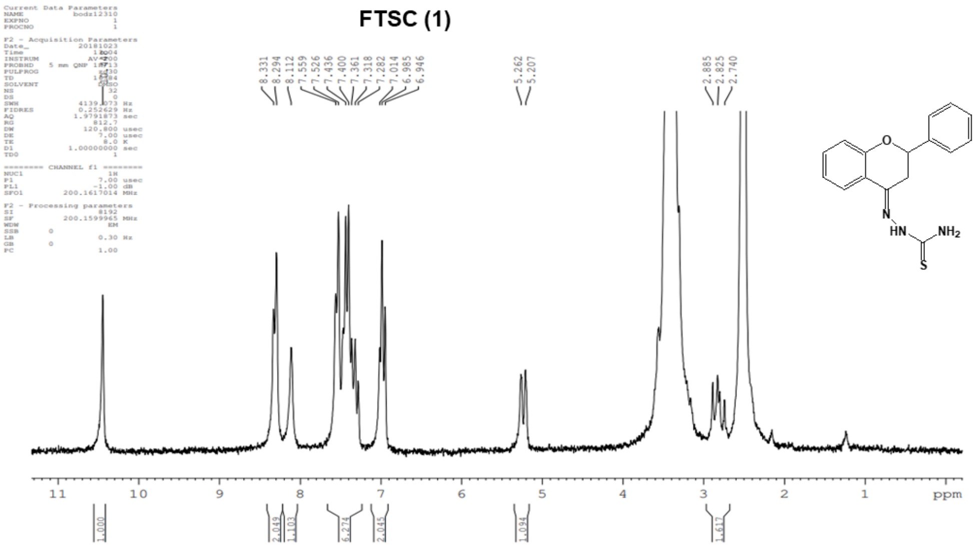

Figure S7. ^{1}H NMR spectrum of compound FTSC (**1**).

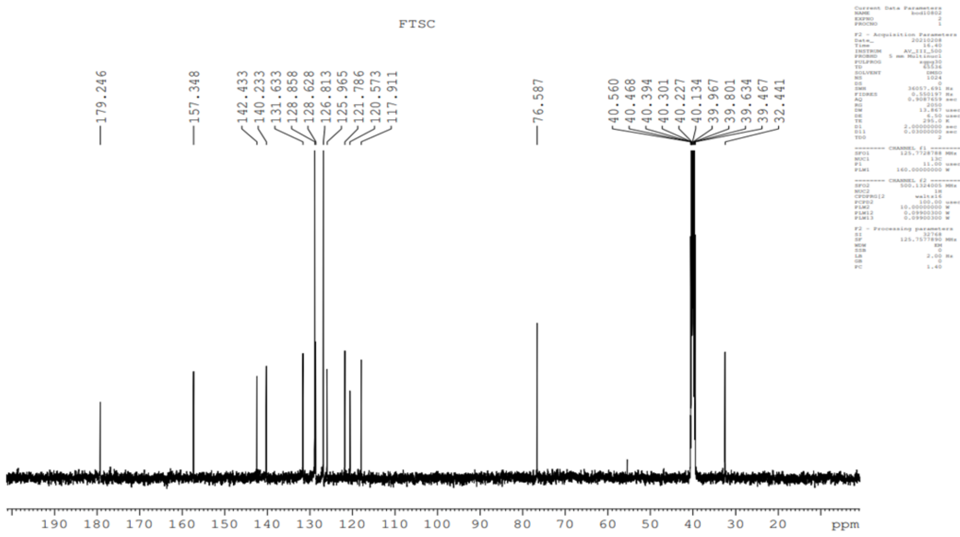

Figure S8. ^{13}C NMR spectrum of compound FTSC (**1**).

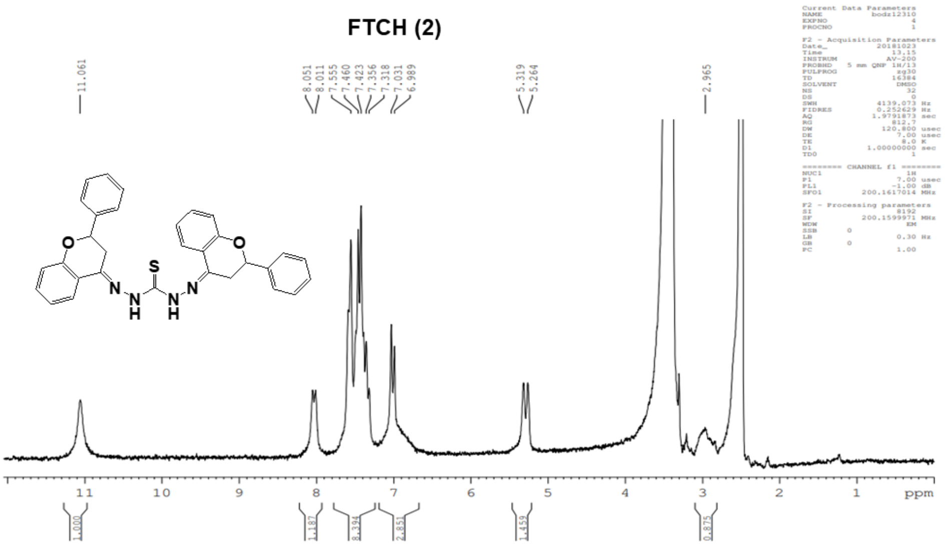

Figure S9. ^{1}H NMR spectrum of compound FTCH (**2**).

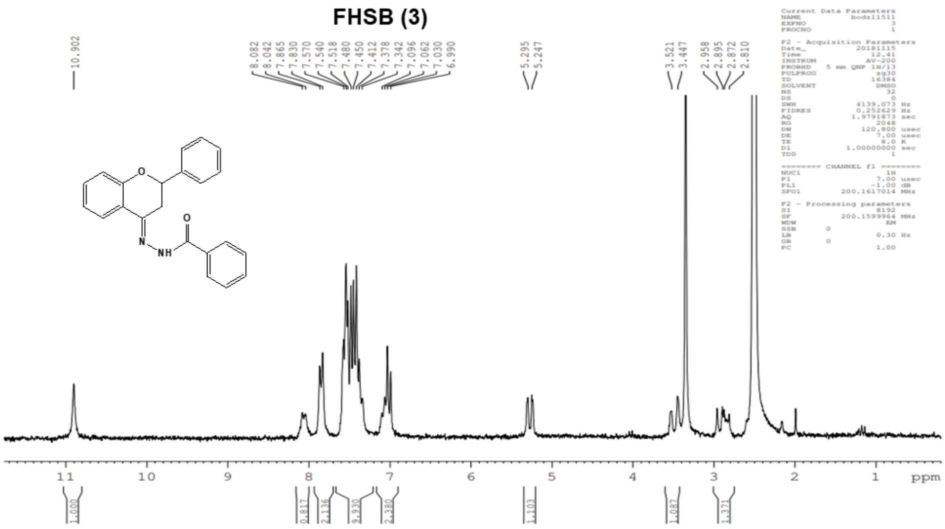

Figure S10. ^{1}H NMR spectrum of compound FHSB (**3**).

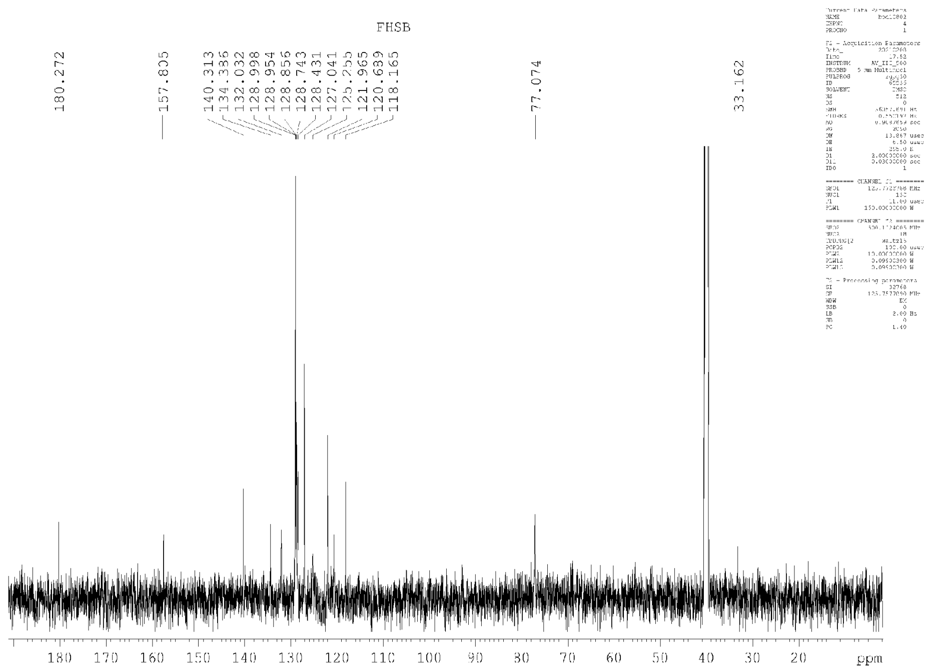

Figure S11. ^{13}C NMR spectrum of compound FIN (**4**).

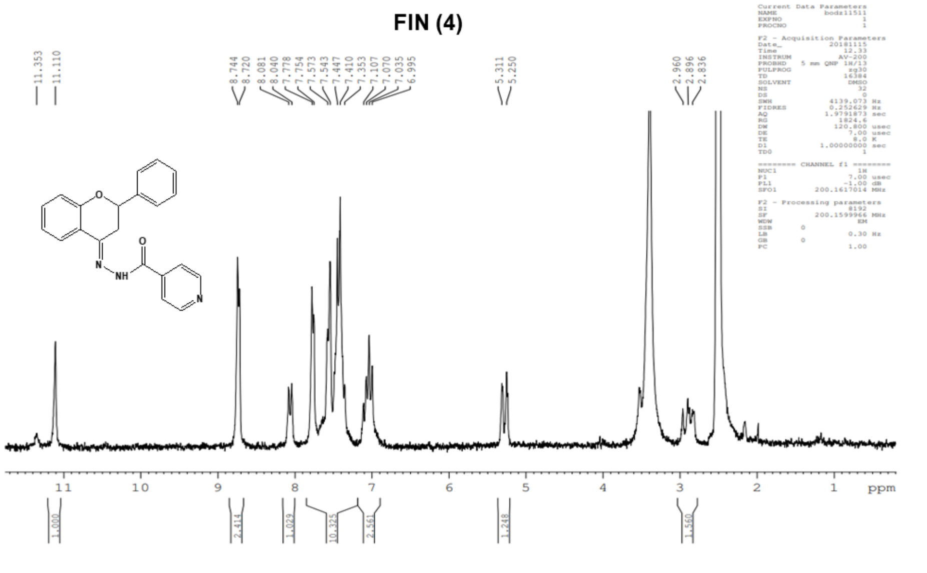

Figure S12. ^{1}H NMR spectrum of compound FIN (**4**).

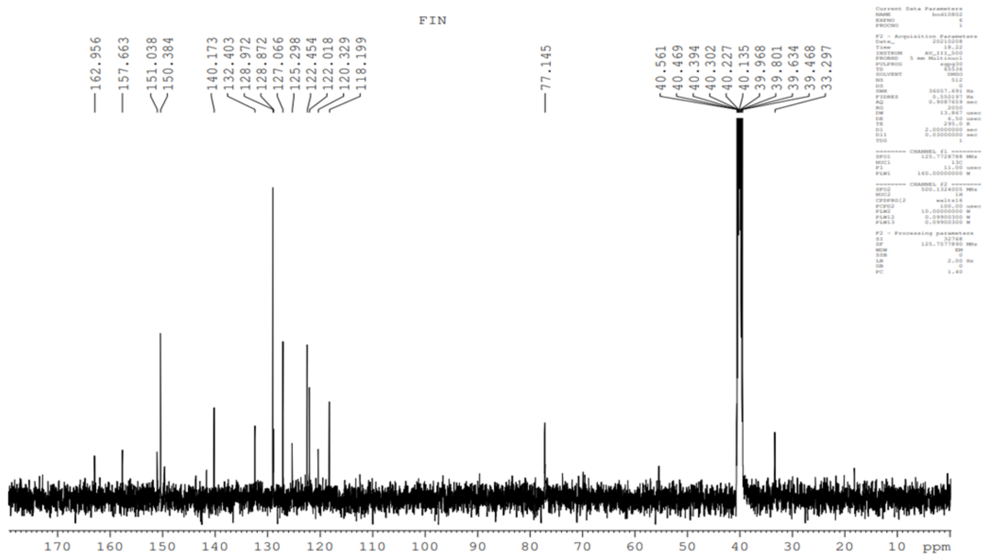

Figure S13. ^{13}C NMR spectrum of compound FIN (**4**).
